# Congenital Microstomia in a Neonate with Impending Respiratory Compromise

**DOI:** 10.1155/2014/739463

**Published:** 2014-12-28

**Authors:** Khoa N. Nguyen, Igor Semenov, Brian Blasiole, Jacob G. Robison, David H. Chi

**Affiliations:** ^1^Department of Anesthesiology, Children's Hospital of Pittsburgh of UPMC, 4401 Penn Avenue, Pittsburgh, PA 15224, USA; ^2^St. Luke's Children's Hospital, Suite 200, 100 East Idaho Street, Boise, ID 83712, USA; ^3^Department of Otolaryngology, Children's Hospital of Pittsburgh of UPMC, 4401 Penn Avenue, Pittsburgh, PA 15224, USA

## Abstract

Microstomia is the term used to describe a reduction in the size of the oral aperture that is severe enough to compromise quality of life, nutrition, and cosmesis. Few cases of congenital microstomia have been reported as most microstomia cases are due to burn injuries. We are presenting a case of a neonate who was found to be in respiratory distress with severe congenital microstomia from no known cause. This case illustrates the rarity of this type of pathologic anatomy as well as the teamwork and tools necessary to treat these patients.

## 1. Introduction

Microstomia is the term used to describe a reduction in the size of the oral aperture that is severe enough to compromise quality of life, nutrition, and cosmesis. Most cases of microstomia reported in the literature are acquired from trauma or accidental electric, thermal, or chemical burns [1]. Few case reports discuss congenital microstomia in a neonate and most are presented in combination with other symptoms as a part of syndromes such as Freeman-Sheldon, Treacher Collins, and Pierre Robin. We present a case report of the anesthetic management of a neonate with congenital microstomia coming for emergent tracheostomy due to respiratory distress.

## 2. Case Description

A 24-hour-old male was referred to the department of otolaryngology for tracheostomy due to respiratory distress and impending respiratory failure. He was born at term by elective cesarean section for prenatally diagnosed ventriculomegaly. At birth, he weighed 3.66 kg and was given APGAR scores of 7 and 8 at 1 and 5 minutes, respectively. He did require oxygen and bag mask ventilation. He was then maintained on nasal CPAP of 7 mm H_2_O and a FiO_2_ of 30%. A prenatal MRI showed he had vermian hypoplasia with associated cisterna magna and severe symmetrical ventriculomegaly with enlarged third ventricle. There were no other abnormalities related to the cardiorespiratory system or craniofacial structure.

**Figure 1:**
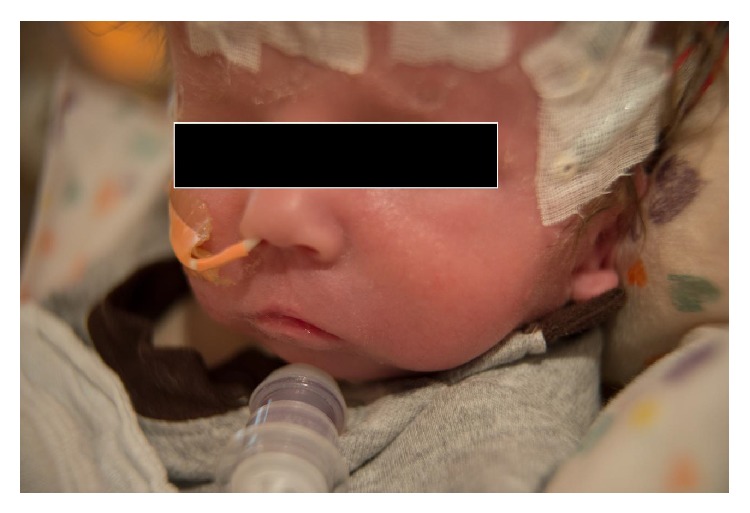
Left frontal view of oral aperture with tracheostomy in place.

He presented to the operating room with respiratory distress consisting of tachypnea and retractions despite the above mentioned ventilatory support. On physical exam he had severe microstomia and retrognathia (Figure 1). He also had a cleft of the second palate. His lungs were clear to auscultation but as mentioned previously he was tachypneic. After placement of standard monitors, he underwent mask induction with oxygen and sevoflurane. Mask ventilation was a challenge, even performed by an experienced anesthesiology attending, due to the retrognathia without any translocation of his mandible to aid in lifting soft tissue. The patient was mask ventilated with an endoscopy mask, which allows for continued positive pressure mask ventilation while providing a site for flexible fiberoptic intubation. The mask has a flexible membrane that a fiberoptic scope can pass through while still maintaining a seal on the patient's face. After instillation of oxymetazoline HCl into the nares, flexible nasolaryngoscopy was performed to evaluate the anatomy, which showed significant tongue base prolapse causing retroflexion of the epiglottis. No other airway abnormalities were noted further in the larynx or trachea. The scope was then removed from the patient and a 3.0 mm ID uncuffed endotracheal tube was then placed over the flexible scope. The scope loaded with the endotracheal tube was maneuvered back into the trachea. Once the appropriate tracheal anatomy was identified the endotracheal tube was advanced into the trachea over the flexible scope. After confirmation of end-tidal CO_2_ and adequate ventilation in both lungs the endotracheal tube was secured. The case then continued with an uneventful tracheostomy and return to the NICU.

## 3. Discussion

Microstomia by itself is a challenge for anesthesiologists performing elective cases. The urgency of a neonate in respiratory distress was adding to the challenge, which made the case more interesting. Most of the case reports about congenital microstomia involve patients with Freeman-Sheldon syndrome otherwise known as craniocarpotarsal dysplasia or whistling face syndrome. All report difficulty with intubation and many report failed intubations. The first case reporting such difficulty was in 1983 involving a father and son with Freeman-Sheldon syndrome [2]. Since then there have been several case reports describing the anesthetic management of patients with Freeman-Sheldon syndrome [3–6] though none of them involve a 2-day-old neonate. The youngest patient reported was 19 months old. Airway management techniques included blind nasal tracheal intubation, awake oral intubation, and fiberoptic intubation as well as laryngeal mask airways. Each patient with different comorbidities presents a unique challenge and one technique may work better than another.

There were several factors in our patient that made the case quite complex. First, the patient was only 2 days old and the combination of small lung volumes, high metabolic rates, and oxygen consumption leaves very little reserve leading to rapid desaturation especially under anesthesia. Furthermore, due to expected factors such as a compliant chest wall and decreased FRC, urgent action was required to secure an airway due to fear of impending respiratory failure. On physical exam, the patient's oral aperture was so small that it could barely be examined even with the smallest digit (Figure 1). A laryngeal mask airway was not ideal due to the existence of the cleft palate as well as the fact that we could not pass a fully deflated and folded-up laryngeal mask airway as has been reported in the past. In addition to the microstomia, the retrognathia and small mandible made mask ventilation difficult despite an experienced pediatric anesthesiologist performing bag mask ventilation.

**Figure 2:**
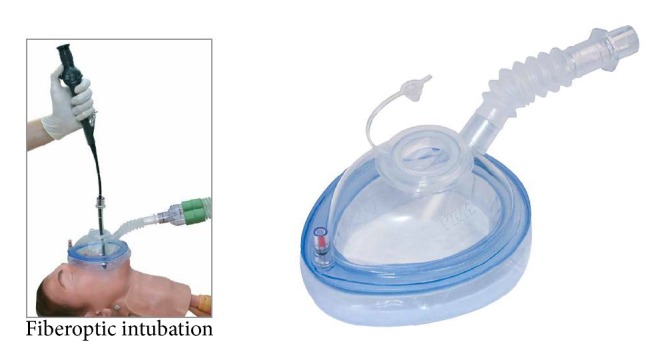
Endoscopy mask (VBM Medizintechnik, Germany).

Fortunately, the anesthesiologist was using an endoscopy mask (Figure 2, VBM Medizintechnik, Germany). It allowed for positive pressure bag mask ventilation while having a small membrane on top of the mask, which could be cut to accommodate a flexible fiberoptic scope without compromising the seal necessary to provide positive pressure. The endoscopy mask, also known as the Patel mask, has been used in adults for over thirty years and is discussed at most pediatric airway courses. Unfortunately, it is not used often enough in clinical practice leaving a generation of anesthesiologists without exposure to mask and its capabilities. A survey of the attending anesthesiologists in our institution showed that most had never seen or heard of such a mask and did not know of its existence. Our institution does stock these masks with different sizes but they were not easy to locate as they are rarely used. One of the purposes of this case report was to improve awareness of these masks that may be useful when most airways are not adequate and positive pressure mask ventilation is required while trying to secure an airway. The otolaryngologists in the room were also impressed with the ease of use and effectiveness of the mask.

In conclusion, this case illustrates the rarity with which patients with such comorbidities present. Despite the rarity of cases, it is reassuring to know that with the appropriate tools and trained staff these patients can be well taken care of. The patient has yet to be diagnosed with a definitive syndrome though he is still being followed up with genetics.
